# A Multi-Level Multiple Contrastive Learning Method for Single-Lead Electrocardiogram Atrial Fibrillation Detection

**DOI:** 10.3390/bioengineering12010044

**Published:** 2025-01-08

**Authors:** Yonggang Zou, Peng Wang, Lidong Du, Xianxiang Chen, Zhenfeng Li, Junxian Song, Zhen Fang

**Affiliations:** 1 Aerospace Information Research Institute, Chinese Academy of Sciences, Beijing 100094, China; zouyonggang20@mails.ucas.ac.cn (Y.Z.); wangpeng01@aircas.ac.cn (P.W.); lddu@mail.ie.ac.cn (L.D.); chenxx@aircas.ac.cn (X.C.); lizhenfeng@aircas.ac.cn (Z.L.); 2 School of Electronic, Electrical and Communication Engineering, University of Chinese Academy of Sciences, Beijing 101408, China; 3 Department of Cardiology, Peking University People’s Hospital, Beijing 100044, China; 4 Research Unit of Personalized Management of Chronic Respiratory Disease, Chinese Academy of Medical Sciences, Beijing 100700, China

**Keywords:** atrial fibrillation, deep learning, electrocardiogram (ECG), contrastive learning (CL)

## Abstract

Atrial fibrillation (AF) is the most common persistent arrhythmia, and it is crucial to develop generalizable automatic AF detection methods. However, supervised AF detection is often limited in performance due to the difficulty in obtaining labeled data. To address the gap between limited labeled data and the requirements for model robustness and generalization in single-lead ECG AF detection, we proposed a semi-supervised contrastive learning method named MLMCL for AF detection. The MLMCL method utilizes the multi-level feature representations of the encoder to perform multiple contrastive learning to fully exploit temporal consistency, channel consistency, and label consistency. Meanwhile, it combines labeled and unlabeled data for pre-training to obtain robust features for downstream tasks. In addition, it uses the domain knowledge in the field of AF diagnosis for domain knowledge augmentation to generate hard samples and improve the distinguishability of ECG representations. In the cross-dataset testing mode, MLMCL had better performance and good stability on different test sets, demonstrating its effectiveness and robustness in the AF detection task. The comparison results with existing studies show that MLMCL outperformed existing methods in external tests. The MLMCL method can be extended and applied to multi-lead scenarios and has reference significance for the development of contrastive learning methods for other arrhythmia.

## 1. Introduction

Atrial fibrillation (AF) is the most common type of sustained arrhythmia [1]. It is defined and characterized by extremely rapid and uncoordinated atrial activities [2]. Atrial fibrillation can significantly increase the risk of developing other high-risk cardiovascular diseases, including stroke [3], systemic embolism [4], vascular dementia [4], heart failure [5], myocardial infarction [6], and sudden cardiac death [7]. Atrial fibrillation usually affects the elderly and initially presents as paroxysmal. Without intervention, paroxysmal AF tends to progress to persistent and permanent AF [2]. Currently, the electrocardiogram (ECG) is the most commonly used monitoring technique for detecting and quantifying the electrical activities of the heart [8]. The occurrence of arrhythmia is usually manifested as the change in ECG morphology and rhythm. For example, the common characteristics of AF on the ECG include irregular RR intervals, the disappearance of P waves, and the appearance of f waves [9]. According to diagnostic conventions, a fibrillation episode lasting at least 30 s can be diagnosed as clinical AF [10]. However, the recognized complexity of ECG is much higher than that of ordinary images, which makes the diagnosis process time-consuming and error-prone. In addition, the quality of ECG diagnosis depends on the professional level of cardiologists, and it may even require multiple experts to resolve differences in decision making. As a result, most of the ECG data collected in the medical field have not been systematically organized and diagnosed. Therefore, it is of great significance to develop efficient and reliable automated detection methods to analyze and interpret ECG recordings.

So far, deep learning (DL) technology has made remarkable progress in the field of automated AF detection. Among them, supervised learning methods dominate current research. Representative deep learning network models include CNN [11,12,13], LSTM [14,15,16,17], Transformer [18,19], the fusion of multiple networks [20,21], etc. These methods have demonstrated excellent performance in multi-lead and single-lead AF detection. Some studies [22] have shown that DL has the potential to reach or even exceed the proficiency level of cardiologists in arrhythmia detection. However, for AF detection, the good generalization performance of supervised learning models depends on a large amount of high-quality annotated ECG data, which is always difficult to obtain. Therefore, it poses a serious problem for AF detection: the AF detection methods based on supervised learning are prone to overfitting in the case of limited annotated data, thus limiting their generalizability.

Considering that multiple instances sharing the same label should share some consistent representations of data that can be learned by the network, AF detection methods based on contrastive learning (CL) [23] attempt to learn this data consistency from unlabeled data to alleviate overfitting, thereby mitigating the impact of limited labeled data and reducing the dependence on labeled data. As a mainstream technique of self-supervised learning (SSL), CL includes two processes: the pre-training task and the downstream task. Among them, the pre-training task usually takes unlabeled data as input, while the labeled data are used for the downstream task. The core idea of this method is to encourage the model to distinguish similar and different data without labels so that the model maps similar samples from the same class closer in the high-dimensional feature space while separating samples from different classes, thus providing meaningful representations for fine-tuning with labeled data in the downstream task and generating more accurate predictions. Specifically, mainstream CL methods bring the representations of positive sample pairs composed of target samples and similar (positive) samples closer in the embedding space while pushing the representations of negative sample pairs composed of target samples and dissimilar (negative) samples farther apart. Therefore, in CL, the selection of positive sample pairs and the design of the corresponding contrastive loss function have received great attention from researchers.

In the field of computer vision, numerous effective CL frameworks such as SimCLR [24], MoCo [25], BYOL [26] widely apply data augmentation to generate positive samples. Among them, SimCLR and its improved versions [27] are the most commonly used frameworks. They take the target sample and the augmented sample as a positive pair, and they regard the target sample and other samples as negative pairs. In the field of time series, some studies attempt to implement augmentation techniques applicable to time series data, such as DTW data augmentation proposed by TimeCLR [28] and random cropping and timestamp masking proposed by TS2Vec [29].

Unlike image data and traditional time series data, there are inherent connections in multiple attribute dimensions among different ECG samples. The data consistency resulting from these internal connections may be beneficial to downstream tasks and can serve as a basis for the selection of positive samples other than data augmentation. For example, TNC [30] conducts subject consistency by taking the time-neighboring samples of the same subject as positive pairs. The CLOCS [31] method further takes into account the samples of different leads and the samples of the same subject to achieve contrastive learning at the channel consistency and subject consistency.

However, despite the certain degree of progress made in the CL methods for ECG, the following problems still exist, which may lead to suboptimal performance in the AF detection task. Firstly, existing CL-based studies usually randomly select negative samples from training data, which means that negative samples may be selected from the samples of the same category as the target sample, resulting in a decline in the quality of representation. Secondly, the downstream task is not used to guide the entire process of positive sample selection in the pre-training task, including data augmentation and the inherent connections of ECG data. Some augmentation methods are unable to generate hard samples with sufficient differences for the downstream task, which is not conducive to learning distinguishable features. Some inherent connections of ECG data are unstable. For example, the paroxysmal nature of AF may cause the ECG data of the same subject to come from multiple distributions, making it impossible for CL to capture the subject consistency. In addition, existing deep learning usually uses data with similar distributions during the training and testing processes, which may exhibit instability in real-world scenarios. The cross-dataset testing method, which evaluates the performance on external datasets that are significantly different from the training data, is becoming an important solution to effectively address this problem.

To address the above issues, we propose a multi-level and multiple contrast learning (MLMCL) solution for AF detection using single-lead ECGs. This method introduces semi-supervised pre-training, which uses both labeled and unlabeled data during the pre-training stage. It constructs robust representations by selecting negative samples based on labels to prevent the decline in representation quality caused by randomly selecting negative samples. To fully tap into the full potential of ECG data, MLMCL performs multiple contrastive learning on the multi-level feature representations extracted by the encoder, which systematically mine the temporal consistency, channel consistency, and label consistency. Among them, the temporal contrastive learning focuses on the representative morphology at a single timestamp, the channel contrastive learning focuses on the invariant information across leads, and the label contrastive learning focuses on the information retained across subjects. In addition, by leveraging the ECG knowledge in the AF-related medical domain, we propose using vertical flipping and T-wave masking to achieve diagnostic region augmentation and non-diagnostic region augmentation, respectively, which together form the domain knowledge augmentation to generate hard samples. We apply MLMCL on the basis of domain knowledge augmentation to learn the generalizable feature representations in ECG, maximizing the potential of both labeled and unlabeled data and reducing the dependence on labeled data. Finally, we conduct pre-training under the cross-dataset testing setting. After obtaining the ECG representations, the model is fine-tuned for AF detection on the labeled dataset. The cross-dataset testing evaluation is carried through linear probing and full fine-tuning to ensure its robustness. Our contributions are as follows:

- This paper proposes a semi-supervised contrastive learning framework that uses both labeled and unlabeled data during the pre-training stage to construct robust representations by selecting negative samples based on labels.
- For AF detection, an MLMCL contrastive learning method is proposed. It performs multiple contrastive learning to extract the temporal consistency, channel consistency, and label consistency on multi-level feature representations, thereby learning generalizable representations.
- By utilizing the knowledge in the AF diagnosis domain, a domain knowledge augmentation combining diagnostic region augmentation and non-diagnostic region augmentation is proposed for generating hard samples to learn distinguishable features.
- The proposed method outperforms existing methods under the cross-dataset testing mode, and the external tests on multiple datasets demonstrate the generalizability of the proposed method.

The remaining parts of this study are arranged as follows. Section 2 introduces the related work. Section 3 outlines the methods used in this study. Section 4 presents the datasets, experiments, and results. Section 5 summarizes this study.

## 2. Related Works

The essence of CL is to mine the common patterns of similar data. According to the requirements of downstream tasks, CL-based methods usually select positive and negative sample pairs in a customized manner. They learn data consistency by maximizing the similarity of the representations of positive sample pairs while minimizing the similarity of the representations of negative sample pairs. Selecting appropriate positive samples is crucial for ensuring the performance of downstream tasks [32]. For example, the SimCLR proposed by Chen et al. [24] defines positive samples as the augmented views of the target sample and directly regards the views from other samples in the current batch as negative samples. Tian et al. [33] use different modal views of the same sample as positive sample pairs. The way of selecting positive samples determines the semantic information of the learned representations. Therefore, it is important to develop a selection strategy of positive sample applicable to ECG.

The CL methods in the ECG field have been studied for extracting effective ECG representations. Some studies directly transfer CL methods from other fields to the ECG field. For example, Mehari et al. [34] directly compared instance-based CL methods (SimCLR, BYOL, and SwAV) and latent forecasting methods (CPC) to demonstrate the feasibility of learning useful representations from 12-lead ECG data through self-supervised learning. Soltanieh et al. [35] used multiple different augmentations and parameters to evaluate the effectiveness of the ECG representations of three contrastive learning methods (SimCRL, BYOL, and SwAV) on out-of-distribution datasets. Zhang et al. [36] adopted a contrastive learning method that manipulates temporal–spatial reverse augmentation to learn ECG representations and explored the impact of different combinations of horizontal flipping (temporal reverse) and vertical flipping (space reverse) in the pre-training stage on the downstream AF detection task. Although these methods have shown improvements over the fully supervised baseline, their selection of positive samples largely depends on data augmentation methods. More ECG contrastive learning methods construct positive samples by combining the data consistencies of the ECG inherent attributes. Such consistencies take various attribute dimensions, and each will be considered in turn, including the consistency of the time, subject, channel, rhythm, morphology, and label.

Temporal Consistency: Temporal consistency, also known as contextual consistency, encourages the feature representations at the same timestamp in different augmented views to be similar. TS2vec [29] proposes contextual consistency in the time dimension of sample representations. It regards the representations of the same timestamp in two augmented views as positive pairs and the representations of different timestamps as negative pairs, focusing on the representative morphology at a single timestamp in the sample.

Subject Consistency: The ECG data of the same subject usually maintains a highly identical pattern within a short period of time, which is the prerequisite for subject consistency. Cheng et al. [37] incorporated a subject-aware condition into the SSL framework to promote the extraction of subject invariance through contrastive loss and adversarial training. Lan et al. [38] proposed an intra-inter subject self-supervised learning (ISL) model for arrhythmia diagnosis. The inter-subject SSL maximizes the subject consistency between different augmented views of the same subject and minimizes the similarity between different subjects to learn the unique representations of differences between different subjects. However, when the ECG data of the same patient comes from multiple distributions, the assumed prerequisite of subject consistency is not satisfied.

Channel Consistency: Different leads data of ECG share the same rhythmic characteristics and represent the same cardiac activity with different waveform morphologies. Channel consistency encourages the learning of invariance across leads, especially rhythmic invariance. Liu et al. [39] proposed a dense lead contrast (DLC) method, which explores the intra-lead and inter-lead invariance through contrastive learning between different leads. In the follow-up work, Liu et al. further [40] proposed a direct lead assignment (DLA) contrastive learning method. In pre-training, DLA simultaneously focuses on the global ECG representation and lead-specific features by performing contrastive learning between multi-lead and single-lead representations, thus improving the quality of single-lead representation. Some studies focused on both the subject consistency and channel consistency of ECG data, such as the 3KG method proposed by Gopal et al. [41] and the CLOCS method proposed by Kiyasseh et al. [31].

Rhythmic Consistency and Morphological Consistency: Liu et al. [42] proposed a morphology–rhythm contrast (MRC) learning framework. MRC performs dual contrastive learning through random beat selection (morphological view) and 0–1 pulse generation (rhythm view), thereby unifying the morphological and rhythmic features. Zhu et al. [43] designed pre-training tasks for intra-period and inter-period representation learning to capture the stable morphological features of a single period and the rhythmic features of multiple periods, respectively. The morphological consistency adopted by these methods [42,43] is a kind of subsequence consistency, which encourages the representation of the time series to be closer to its sampled subsequence. However, the representation of single-period subsequence features usually cannot reflect the overall morphological features. Due to the loss of information, subsequence consistency may not be applicable to ECG data.

Label Consistency: The label consistency representation is the goal of downstream tasks. Incorporating label information into contrastive learning can provide good initialization parameters for downstream tasks. The supervised CL proposed by Khosla et al. [44] extended CL from the self-supervised domain to the full-supervised domain. The proposed supervised contrastive loss function (SupCon), by introducing label information, makes samples belonging to the same class cluster more closely in the embedding space, while samples of different classes are pushed away from each other. On this basis, Le et al. [45] applied supervised contrastive learning to the classification of multi-lead ECG arrhythmias.

The above studies have demonstrated that using CL methods for ECG representation learning can achieve performance comparable to or even surpassing that of fully supervised methods. Summarizing the current research, existing methods mainly consider the consistency of attributes such as the time, channel, subject, rhythm, morphology (subsequence), and label of the ECG. However, existing studies usually only consider one or two attribute consistencies of ECG data and overlook the degree of matching of these strategies with the downstream task data, such as subsequence consistency. Few studies have proposed customized data augmentation methods for downstream tasks. Additionally, existing methods usually only use unlabeled data or only use labeled data for pre-training. These limitations result in the inability of existing methods to fully tap into the full potential of ECG data. To overcome these limitations, we develop a CL method for AF detection by leveraging the ECG data consistencies of multiple attribute dimensions such as time, channel, and label, as well as AF domain knowledge augmentation methods. We do not specifically implement rhythm consistency, since it has already been achieved in channel consistency and diagnostic region augmentation. Due to the presence of a large number of paroxysmal samples in AF records, which may cause the data of the same subject to have different distributions, we abandon the subject consistency strategy in AF detection.

## 3. Method

**Figure 1:**
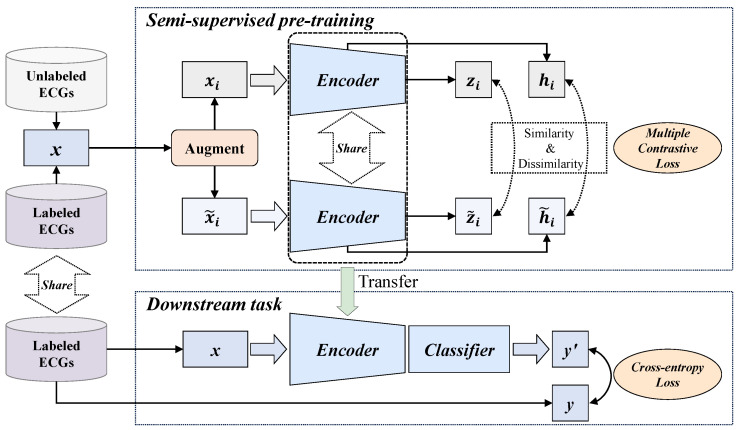
The overall flow of the proposed MLMCL algorithm.

The overall process of the proposed MLMCL algorithm is shown in Figure 1, which includes two parts: the semi-supervised pre-training task and the downstream task. In the pre-training part, multiple contrast learning is performed on multi-level representations of labeled and unlabeled data, aiming to learn the general representations of single-lead ECGs. The downstream task is the AF detection in single-lead ECG. The MLMCL method contains three key components: domain knowledge augmentation, a multi-level encoder, and multiple contrastive losses. The following provides a more detailed description from three aspects: data augmentation, contrastive learning pre-training, and the downstream task.

### 3.1. Domain Knowledge Augmentation

Data augmentation is a crucial part for the success of CL methods. It does not need to utilize the inherent connections (such as label categories) of different sample data to obtain similar positive samples. Instead, it only needs to directly transform a single sample to generate similar views. When the data augmentation transformation operators are applied to each ECG instance, the guarantee for extracting efficient representations is to maintain the invariance of important information in the ECG records. However, sometimes series data augmentation methods produce less difference between the augmented sample and the original ECG sample, making it impossible to generate reliable “hard” samples to help the model locate the key invariant features related to downstream tasks.

In ECG, arrhythmia may change the ECG rhythm and the morphology of specific regions, and each type of arrhythmia has distinguishable characteristic patterns. We attempted to utilize the medical domain knowledge of ECG diagnosis to address the issue of hard sample generation [46]. The proposed domain knowledge augmentation is a method that combines diagnostic region augmentation and non-diagnostic region augmentation. Both the diagnostic and non-diagnostic region augmentation modify the signal values in the corresponding regions to generate “hard” samples for achieving robust learning. The aim is to transform the waveform of the diagnostic/non-diagnostic regions to a large extent so that it is distinguishable from the original sample and other ECG categories, thereby explicitly guiding the model to learn distinguishable features.

**Figure 2:**
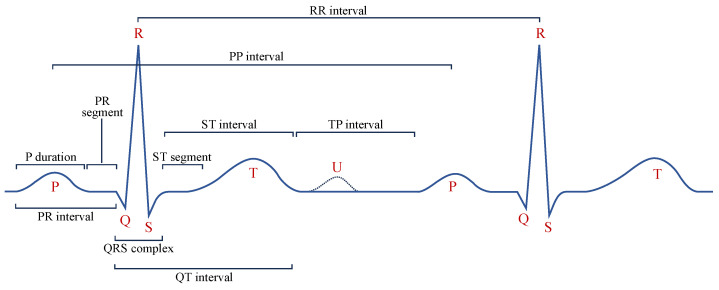
The classical ECG waveform and crucial segments with measurement points.

Diagnostic Region Augmentation: As shown in Figure 2, the key diagnostic regions of AF lie in the P-wave and QRS-wave regions. Considering that the positive and negative morphologies of P wave/QRS wave are symmetrical in the vertical direction, we thus adopt vertical flipping for diagnostic region augmentation. Vertical flipping will create a sufficiently large difference between the original ECG sample and the augmented sample, without causing confusion between the P wave and f wave. The vertical flipping augmentation of the raw ECG signal x can be represented as $\tilde{x}=-x$.

Non-Diagnostic Region Augmentation: For the non-diagnostic regions of AF, considering that the morphology and position of the T wave are not the key factors for diagnosing AF, we adopt the method of T-wave masking to reduce the interference of the T-wave part on the extraction of diagnostic features, making the model focus more on the diagnostic regions such as the P wave and QRS wave. T-wave masking sets the specific ST interval in the single-lead ECG signal to a fixed value. Specifically, its implementation includes two steps. First, the QRS-wave region of each heartbeat needs to be identified on the entire ECG record, since the QRS wave is the most distinctive wave region and is widely used as a reference for locating other characteristic wave regions. Second, the *c*% number of the ST intervals in the single-lead ECG sample are set to a fixed value. Usually, the T wave appears within the range of 300 ms after the R wave. Considering that the duration of the QRS wave is usually 80 ms to 100 ms, we achieved T-wave masking by setting the region from 50 ms to 300 ms after the R wave to a fixed value. In the experiments, a typical value for the masking parameter is c=50.

**Figure 3:**
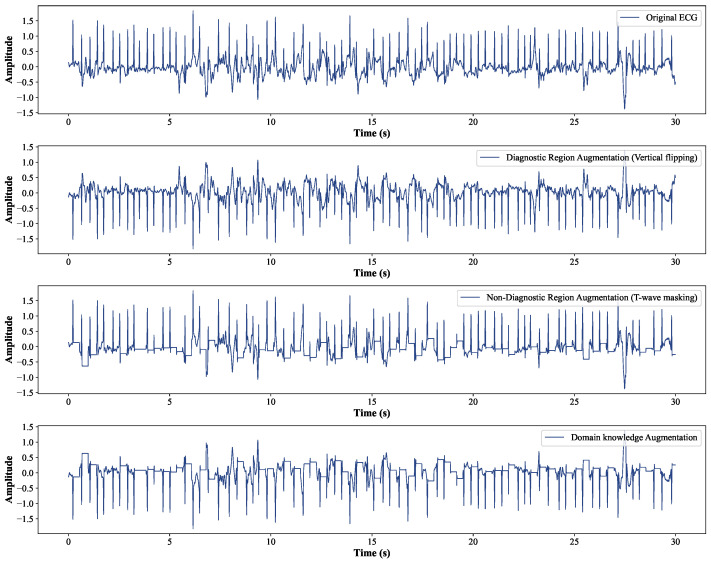
The diagram of domain knowledge augmentation.

Typically, contrastive learning methods use two augmented variants with different strengths to improve the robustness of the learned representations [47]. In this paper, the weak augmented variant directly uses the pre-processed data without transforming the original ECG sample, since the ECG samples are not augmented in the downstream task. The strong augmented variant adopts the domain knowledge augmentation strategy to generate “hard” samples. A typical implementation of domain knowledge augmentation is shown in Figure 3. In fact, we applied diagnostic region augmentation on top of non-diagnostic region augmentation with a 50% probability to achieve domain knowledge enhancement. Given a raw single-lead ECG sample $x_{i}$, augmentation produces two views that can be represented as $x_{i}$ and ${\tilde{x}}_{i}$, respectively. These views are passed to the encoder to extract their high-dimensional latent representations.

### 3.2. Multi-Level Multiple Contrastive Learning Pre-Training

The proposed MLMCL is a semi-supervised contrastive learning method for single-lead ECGs, which can learn ECG representations from both labeled and unlabeled data simultaneously. Each single-lead ECG $x\in R^{T_{0}\times 1}$ is regarded as single-channel 1D data with a sequence length of $T_{0}$. Given a labeled dataset $D_{L}={\left\{x_{i},y_{i}\right\}}_{i=1\ldots M}$ that contains *M* instance/label pairs and an unlabeled dataset $D_{U}={\left\{x_{i}\right\}}_{i=1\ldots N}$ that contains *N* instances, the goal of the MLMCL pre-training is to learn an encoder to extract effective representations $z_{i}\in R^{D_{z}}$ relevant to the downstream task from each $x_{i}$.

During the pre-training stage, two augmented copies of the same sample are fed into a multi-level encoder to obtain the intermediate hidden representations and the output encoder representations, and data consistency in multiple attribute dimensions on the multi-layer representations is encouraged. The temporal contrastive loss is calculated on the hidden representations, and the channel contrastive loss and label contrastive loss are calculated on the encoder representations. By utilizing the inherent data consistency in multiple dimensions through contrastive learning, the encoder is optimized with multiple contrastive losses to help learn a representation that is both representative and generalizable.

**Figure 4:**
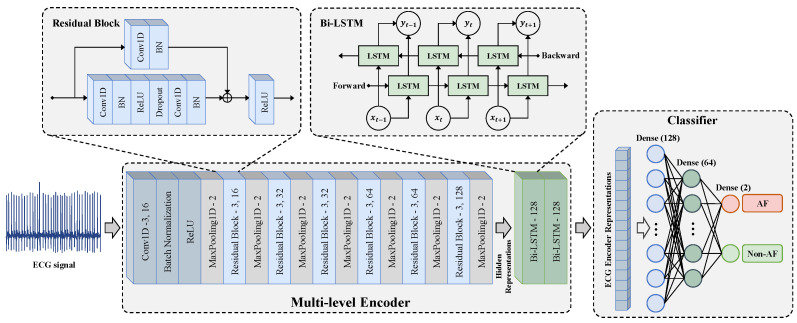
The encoder architecture used for pre-training and the classifier architecture used for fine-tuning.

As shown in Figure 4, the multi-level encoder consists of two parts, including a convolutional feature extraction network f(·) and a temporal–spatial feature fusion network g(·). The convolutional feature extraction network adopts a residual block design. It extracts the context representation of each sample through one input convolutional layer and six residual blocks. It maps $x_{i}$ to a high-dimensional latent space to obtain the hidden representation $h_{i}=f\left(x_{i}\right)\in R^{T\times D_{h}}$. The hidden representation $h_{i}=\left\{h_{i,1},h_{i,2},\ldots ,h_{i,T}\right\}$ contains *T* representation vectors in the time dimension. The feature vector $h_{i,t}\in R^{D_{h}}$ at the *t*-th timestamp is a representation vector with $D_{h}$ dimensions and is used to calculate the temporal contrastive loss.

The temporal–spatial feature fusion network g(·) is constructed using the Bidirectional Long Short-Term Memory Network (Bi-LSTM) to extract the temporal-spatial features of ECG. The g(·) learns from the hidden representation $h_{i}$ to obtain the representation $r_{i}=g\left(h_{i}\right)\in R^{T\times D_{z}}$, which also contains T representation vectors in the time dimension. Since the output of the last time step of the LSTM network integrates the temporal–spatial information of $h_{i}$ in order to reduce the amount of computation, the output $z_{i}=r_{i,T}$ of the last time step is taken as the output encoder representation. The output $z_{i}$ of the encoder is normalized onto the unit hypersphere in $R^{D_{z}}$, which makes it possible to use the inner product to measure the distance in the latent space.

Unlike the models with the SimCLR architecture, we adopted a projector-free design. The experimental results (Section 4.4.3) show that our method did not lead to a performance decline without using a projector. In fact, the absence of an additional projector could further reduce the pre-training parameters and time consumption.

#### 3.2.1. Temporal Contrastive Learning

Contrastive learning generally assumes that the representation of an augmented sample will carry information similar to that of the corresponding original sample. Analogously, their representation vectors at the same timestamp will also carry similar context information, especially for high-dimensional latent representations extracted by convolutional networks because of the same receptive field and the same network parameters. In order to learn discriminative representations that change over time, we chose to learn temporal consistency. For two augmentations of the same sample, representation vectors with the same timestamp are regarded as positive pairs, and representation vectors from different timestamps are treated as negative pairs [29]. Temporal contrastive learning helps the encoder focus on the representative features at a single timestamp. Since temporal consistency does not rely on labels, temporal contrastive learning was performed on both labeled and unlabeled data.

For a given ECG sample $x_{i}$ and its augmentation ${\tilde{x}}_{i}$, the convolutional feature extraction network in the encoder extracts their respective hidden representations as $h_{i}=f\left(x_{i}\right)$ and ${\tilde{h}}_{i}=f\left({\tilde{x}}_{i}\right)$, respectively. To capture the temporal consistency, the positive pair is $\left(h_{i,t},{\tilde{h}}_{i,t}\right)$, and the negative pairs are $\left(h_{i,t},h_{i,t^{\prime }}\right)$ and $\left(h_{i,t},{\tilde{h}}_{i,t^{\prime }}\right)$. In practice, the contrastive loss is calculated within a mini-batch of data. In a batch with *B* raw ECG samples, the temporal contrastive loss [29] is defined as

(1) $$L_{tCL}=-\frac{1}{B}\sum_{i=1}^{B}\frac{1}{T}\sum_{t=1}^{T}\log\frac{\exp\left(h_{i,t}⊙{\tilde{h}}_{i,t}/\tau \right)}{\sum_{t^{\prime }=1}^{T}\left(\exp\left(h_{i,t}⊙{\tilde{h}}_{i,t^{\prime }}/\tau \right)+1_{\left\{t^{\prime }\neq t\right\}}\exp\;\left(h_{i,t}⊙h_{i,t^{\prime }}/\tau \right)\right)}$$

where $1_{\left\{t^{\prime }\neq t\right\}}$ is an indicator function, which equals 1 when the condition $t^{\prime }\neq t$ is satisfied and equals 0 otherwise. The function exp(·) is the exponential function and the function log(·) is the natural logarithm function. The symbol $\tau \in R^{+}$ is a scalar temperature parameter used to adjust the slope of the loss function. The symbol ⊙ represents the inner product operation and is used to calculate the cosine similarity between two vectors. The cosine similarity of vectors u and v is defined as $u⊙v=u^{T}v/\left(∥u∥∥v∥\right)$.

#### 3.2.2. Channel Contrastive Learning

The 12 lead signals in the same ECG can be thought of as natural augmentation of each other [31], since multiple lead ECG signals collected simultaneously will reflect the same cardiac activity, and they are associated with the same class. Although some arrhythmias affect specific parts of the heart so that they can only be detected by a few leads, the irregular rhythm of AF is special and can be observed in all leads. Minimizing the inter-lead differences helps to discover the rhythm invariance among the leads [48]. Therefore, we utilize the invariance of different leads for channel contrastive learning.

For a given ECG sample $x_{i}$, its time-aligned sample of other leads can be represented as $x_{i}^{\prime }$. The samples $x_{i}$ and $x_{i}^{\prime }$ are input into the encoder to obtain their respective encoder representations $z_{i}$ and $z_{i}^{\prime }$. To capture the channel consistency, the encoder representation $z_{i}$ should be close to $z_{i}^{\prime }$, and conversely, away from the representation $z_{j}$ and $z_{j}^{\prime }$ of any other different samples. Specifically, the positive pair is $\left(z_{i},z_{i}^{\prime }\right)$, and the negative pairs are $\left(z_{i},z_{j}\right)$ and $\left(z_{i},z_{j}^{\prime }\right)$. In a batch with *B* initial ECG samples, the channel contrastive loss [39] is defined as

(2) $$L_{cCL}=-\frac{1}{B}\sum_{i=1}^{B}\log\frac{\exp\left(z_{i}⊙z_{i}^{\prime }/\tau \right)}{\sum_{j=1}^{N}\left(\exp\left(z_{i}⊙z_{j}^{\prime }/\tau \right)+1_{\left\{j\neq i\right\}}\exp\left(z_{i}⊙z_{j}/\tau \right)\right)}$$

where the indicator function $1_{\left\{j\neq i\right\}}$ equals 1 when the condition j≠i is satisfied and equals 0 otherwise.

#### 3.2.3. Label Contrastive Learning

In self-supervised contrastive learning, the negative samples of the target sample are composed of samples randomly selected from the mini-batch data. When multiple negative samples and the target sample have the same label, the contrastive loss may push samples of the same class further apart. For downstream supervised tasks, this may lead to a decline in the quality of the representation. Therefore, label consistency was introduced to solve this problem. Label consistency is beneficial for learning domain-adaptive representations of certain diseases across patients or even datasets because it indicates that samples with the same label should exhibit shared patterns even if they are collected from different subjects in different ways. Here, we adopted semi-supervised learning in pre-training to introduce labeled data and label contrastive loss.

In a batch with *B* initial ECG samples, the positive samples of the target sample $x_{i}$ will be generalized to any number of samples $x_{p}$ that have the same label as the target sample. Different from the self-supervised contrastive loss, the label contrastive loss contrasts the set of all samples with the same class label with the remaining samples of other classes in the batch. The calculation formula of label contrastive loss [44] is as follows:

(3) $$L_{lCL}=-\frac{1}{B}\sum_{i\in I}\frac{1}{|P_{i}|}\sum_{p\in P_{i}}\log\frac{\exp\left(z_{i}⊙z_{p}/\tau \right)}{\sum_{j\in A_{i}}\exp\left(z_{i}⊙z_{j}/\tau \right)}$$

In the formula, $A_{i}=\left\{I\setminus \left\{i\right\}\right\}$ is the set of indices in the batch index I=1,2,…,B that do not include *i*, $P_{i}=\left\{p\in A_{i}:y_{p}=y_{i}\right\}$ is the set of sample indices in the batch that have the same label as sample *i*, and $|P_{i}|$ is the number of its elements.

#### 3.2.4. Multiple Contrastive Loss

**Figure 5:**
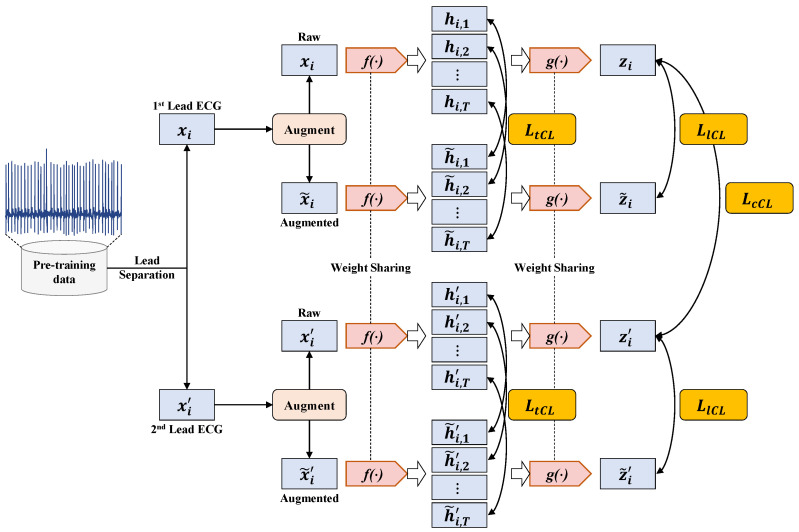
The calculation process of multiple contrastive loss.

The calculation process of the multiple contrastive loss used in MLMCL is shown as Figure 5. The multiple contrastive loss $L_{CL}$ consists of three loss terms. The temporal contrastive loss and the label contrastive loss help to learn robust representations that are invariant to transformations, while the channel contrastive loss encourages the encoder to learn lead-invariant representations. To sum up, the multiple contrastive loss of the MLMCL method is as follows:

(4) $$L_{CL}=\lambda_{1}L_{tCL}+\lambda_{2}L_{cCL}+\lambda_{3}L_{lCL}$$

where $\lambda_{1},\lambda_{2},\lambda_{3}\in \left[0,1\right]$ are hyperparameters that adjust the scale of each loss and satisfy $\lambda_{1}+\lambda_{2}+\lambda_{3}=1$. For the labeled dataset, three contrastive losses were adopted. For the unlabeled data, only temporal contrastive loss and channel contrastive loss were adopted, that is, $\lambda_{3}$ was set to 0.

### 3.3. Fine-Tuning for AF Detection Task

After the MLMCL pre-training is completed, the encoder composed of the convolutional feature extraction network f(·) and the temporal–spatial feature fusion network g(·) are passed to the downstream AF detection task as a feature extractor. During fine-tuning, in order to use the pre-trained encoder for classification, the encoder is fine-tuned and a classifier is trained. The classifier is usually designed as a multi-layer perceptron (MLP) with a hidden layer, and ReLU is the activation function used between each fully connected layer. As shown in Figure 4, the classifier used in the downstream task was designed with three fully connected layers, and a batch normalization layer was added before the activation function to stabilize the training. The cross-entropy loss was used for the supervised training of AF detection:

(5) $$L_{CE}=-\frac{1}{B}\sum_{j=1}^{B}\sum_{i=1}^{C}\left(y_{i}logy_{i}^{\prime }+\left(1-y_{i}\right)\log\left(1-y_{i}^{\prime }\right)\right)$$

Among them, *C* is the number of categories, $y_{i}$ is the *i*-th element of the true label y, and $y_{i}^{\prime }$ is the *i*-th element of the prediction result $y^{\prime }$.

## 4. Experiments and Results

### 4.1. Databases and Data Preprocessing

In this work, four public databases from Physionet [49] were used to evaluate the proposed method. There is no subject overlap among these databases. The specific introductions are as follows:

- MIT-BIH Atrial Fibrillation Database (AFDB) [50]: It consists of long-term ECG recordings of 23 human subjects with AF (mostly paroxysmal). Each recording contains two different lead ECG signals with unspecified lead names and lasts for 10 h with a sampling rate of 250 Hz. Rhythm annotation files and heartbeat annotation files are provided separately. The rhythm annotation files are manually prepared, and most of the heartbeat annotation files are obtained by an automatic detector without manual correction.
- The 4th China Physiological Signal Challenge 2021 (CPSC2021) [51]: This database has publicly released two training sets, which altogether include 1406 dual-lead recordings from 105 patients. These recordings have been extracted from the ECG readings of 49 AF patients (23 paroxysmal AF patients) and 56 non-AF patients (usually including other abnormal and normal rhythms). The provided annotations include heartbeat annotations, rhythm annotations, and diagnoses of the global rhythm. Each recording consists of lead I and lead II signals, with a sampling frequency of 200 Hz, and the duration of each recording is not fixed.
- Long-Term AF Database (LTAFDB) [52]: It includes long-term ECG recordings of 84 subjects with paroxysmal or persistent AF. Each recording contains two simultaneously recorded ECG signals with a sampling frequency of 128 Hz, and the recording duration usually ranges from 24 to 25 h.
- MIT-BIH Arrhythmia Database (MITDB) [53]: It contains 48 half-hour dual-channel ECG recordings from 47 subjects, and the recordings are digitized at 360 Hz. Two or more cardiologists independently annotated each recording, and the reference annotations for each heartbeat were given after resolving differences. The lead names of the two ECG signals in each ECG recording are not fixed, mainly leads II and V1, and a few are leads V2, V4, and V5.

The detailed information of the four open-source datasets is summarized in Table 1. The channels of leads I and II were preferentially used when lead information was available. If it was unavailable, the channel similar to lead II among the two channels was selected for analysis.

Before the experiment, the ECG recordings were resampled to 128 Hz to ensure a uniform sampling rate. A band-pass filter with a frequency range of 0.5–40 Hz was used to remove the baseline drift and high-frequency noise in the ECG signals. Each recording was segmented into 30 s ECG segments for analysis. The parts shorter than 30 s were discarded. Each ECG segment was normalized using the z-score method. According to the annotations, each segment was labeled as AF or non-AF. Among them, all rhythms except AF and AFL were labeled as non-AF rhythms. To remove the segments containing severe noise, the beat signal quality index (bSQI) [53] of each ECG segment was used to evaluate the signal quality. The segments with bSQI < 0.8 were excluded from the analysis in this paper. In addition, we reserved the segments of another simultaneously collected lead for each ECG segment for subsequent experiments. The detailed segmentation information of the databases is shown in Table 2.

### 4.2. Experimental Settings

#### 4.2.1. Evaluation Paradigms

It is worth noting that the quality of the representations from semi-supervised pre-training should be evaluated by the downstream AF detection task. Better performance of AF detection indicates higher quality of ECG representations. To evaluate the performance, the accuracy (Acc), macro sensitivity (Sen), macro precision (Pre), and macro F1-score (F1) were used as the metrics of classification performance. All these metrics range from 0 to 1. The larger the value, the better the performance.

#### 4.2.2. Parameter Settings

For the input data, normalized single-lead ECGs with a sampling frequency of 128 Hz and a time length of 30 s were used for both pre-training and fine-tuning. In the pre-training stage, the length of the hidden representation vector was $D_{h}=128$, and the length of the encoded representation vector was $D_{z}=256$. In addition to τ=1.0 for temporal contrastive loss, τ=0.1 was used as the temperature hyperparameter for all other contrastive losses. For the labeled data, the loss coefficients adopted $\lambda_{1}=\lambda_{2}=\lambda_{3}$, while for the unlabeled data, the loss coefficients adopted $\lambda_{1}=\lambda_{2}$. The entire pre-training process lasted for 40 epochs, and the batch size of the pre-training data was 256. The Adam optimizer was used with an initial learning rate of 0.001. The StepLR was adopted to adjust the learning rate, and the learning rate decayed to 0.8 of the original value every three epochs, ensuring the stability of the entire optimization process. In the fine-tuning stage, the Adam optimizer was also used for parameter optimization, and the initial learning rate $\eta_{base}$ was set to the same as in the pre-training stage. The batch size for fine-tuning was 512, and the number of iterative epochs $T_{max}$ was 30. The formula for the learning rate change with training epochs is

(6) $$\eta =\eta_{base}/{\left(1+10\times T_{cur}/T_{max}\right)}^{2}$$

where $T_{cur}$ represents the current training epoch.

#### 4.2.3. Implementation

All models were implemented based on PyTorch (v1.12.1). With the support of the Intel (R) Xeon (R) E5-2640 CPU and the NVIDA GeForce RTX 3090 GPU in terms of hardware configuration, Python (v3.9.15) was used to implement the pre-training, fine-tuning, and testing of the proposed MLMCL method.

In this work, we mainly adopted the cross-dataset testing mode, which tests with external datasets that have not been seen in the model training phase. The cross-dataset testing follows the inter-patient paradigm. The CPSC2021 dataset was used as the labeled training set for pre-training and downstream task training, because it has the largest number of individual subjects, a relatively balanced data distribution, and sufficient training samples. The AFDB and LTAFDB were respectively used as external independent test sets. Each test dataset was not used in the pre-training and fine-tuning processes but only for evaluation. During their testing, the remaining datasets were added to the pre-training as unlabeled data.

We tried linear probing and full fine-tuning in the training of downstream AF detection model. During training, linear probing freezes the encoder parameters and only updates the classifier parameters to perform AF detection. It is usually used to evaluate the quality of the learned representations for that downstream results completely depend on the pre-trained encoder. In contrast, full fine-tuning adjusts both the encoder and the classifier simultaneously to adapt to the AF detection task. In the full fine-tuning mode, pre-training is equivalent to providing more effective initial parameter values.

#### 4.2.4. Baselines

To compare with the proposed MLMCL method, we conducted four baseline methods. (1) Fully supervised (FS): Supervised training was started on randomly initialized encoders and classifiers. (2) Fully supervised with data augmentation (FS + DA): Domain knowledge augmentation was introduced on the basis of full supervision. (3) SimCLR [24]: As a classical framework of CL, SimCLR forms positive samples of target sample only through data augmentation and takes other samples of the same batch as negative samples. (4) T-S [36]: Temporal reverse, spatial reverse, and temporal–spatial reverse were performed on the original signals, and then pre-training was completed by classifying four signals, including the original signal.

It is worth noting that these baseline methods used the same encoder architecture and datasets as the proposed MLMCL method for comparison.

### 4.3. Results

In this section, we evaluated the effectiveness of the proposed MLMCL method and compared it with various baseline methods on the AF detection task.

#### 4.3.1. Linear Probing

Linear probing aims to assess the quality of representations. Table 3 shows the linear probing results of AF detection on the AFDB and LTAFDB databases. In the linear probing mode, the fully supervised method used a randomly initialized encoder and did not have any prior knowledge about ECG information. The results show that MLMCL outperformed the fully supervised method by 47.54% and 33.54% in terms of Acc and by 50.54% and 33.61% in terms of F1-score on AFDB and LTAFDB, which verifies the effectiveness of the pre-training. Similarly, MLMCL also obtained results that were significantly higher than those of the fully supervised method with data augmentation. Compared to SimCLR and T-S that used self-supervised training, MLMCL achieved better performance. Overall, the Acc and F1-scores of MLMCL on all external test sets were higher than those of all the baselines, which indicates that MLMCL is able to provide high-quality representations.

#### 4.3.2. Full Fine-Tuning

Full fine-tuning allows fine-tuning of the entire network parameters. Table 4 presents the full fine-tuning results of MLMCL and multiple baselines. Compared with the fully supervised training with a randomly initialized encoder, the performance of the model pre-trained by MLMCL was greatly improved, indicating that it provides a more effective network initialization. In addition, MLMCL consistently outperformed the fully supervised method with data augmentation on AFDB and LTAFDB, which shows that MLMCL pre-training is more helpful for the encoder to extract key features from the augmented data. Overall, compared to fully supervised training, MLMCL can achieve better performance by simultaneously utilizing labeled and unlabeled data, alleviating the annotation burden in practical applications.

**Figure 6:**
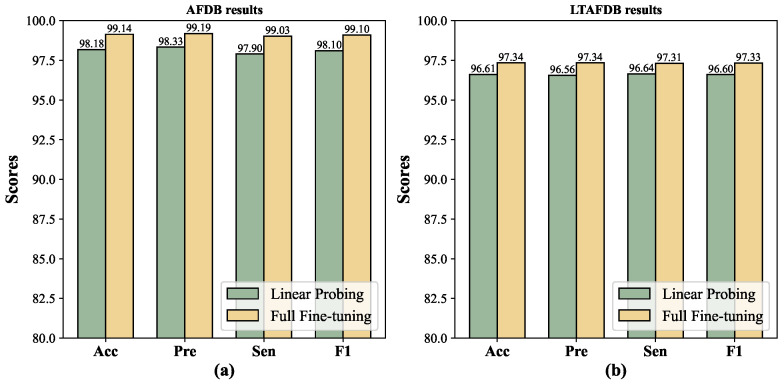
The comparison between linear probing and fully fine-tuning. (**a**) AFDB results. (**b**) LTAFDB results.

In addition, the Acc and F1-score of MLMCL exceeded those of the best baseline SimCLR by 2.59% (96.55% vs. 99.14%) and 2.68% (96.42% vs. 99.10%), respectively, on AFDB. On the LTAFDB dataset, the Acc and F1-score of MLMCL exceeded those of the best baseline T-S by 1.10% (96.24% vs. 97.34%) and 1.11% (96.22% vs. 97.33%), respectively. Compared to other contrastive learning baseline methods, MLMCL had a consistent advantage on different test sets, indicating that it has good stability. As shown in Figure 6, the fully fine-tuning method generally achieved better performance than linear probing.

### 4.4. Ablation Study

In this section, we investigate the necessity and effectiveness of the key components and settings in the proposed method for AF detection, including domain knowledge augmentation, multiple contrastive loss, and the design without a projector. The following ablation experiments all adopt the results of full fine-tuning.

#### 4.4.1. Data Augmentation

The MLMCL method uses ECGs processed by domain knowledge augmentation, not just the raw ECG records. To evaluate the effectiveness of domain knowledge augmentation on AF detection, we separately evaluated the performance when the diagnostic region augmentation (vertical flipping) and non-diagnostic region augmentation (T-wave masking) were used alone. In addition, the following data augmentation methods commonly used for time series [35] were compared:

- Gaussian noise: A Gaussian noise signal n(t) with a mean of 0 was added to the ECG sample x(t) to obtain the augmented signal $\tilde{x}\left(t\right)=x\left(t\right)+n\left(t\right)$. A random standard deviation of noise in the range of [0.01,0.1] was adopted in the experiment.
- Baseline wander: A low-frequency sinusoidal wave with frequency $f_{w}$ and maximum amplitude $S_{w}$ were added to the ECG sample to achieve baseline wander. In the experiment, the range of $f_{w}$ was [1/30 Hz, 1/10 Hz], and the range of $S_{w}$ was [0.5,0.6].
- Channel scaling: The ECG sample was multiplied by a scaling factor *S* to achieve scaling. A random scaling factor *S* in the range of [0.1,5] was tested in the experiment.
- Horizontal flipping: Horizontal flipping was considered as temporal reverse, which can be expressed as $\tilde{x}\left(t\right)=x\left(T_{0}-t+1\right)$, where $t=1,2,\ldots ,T_{0}$.
- Time warping: First, the ECG sample x(t) was divided into *w* segments. Next, for each segment, half of the areas were randomly selected to stretch them by a factor of *r*% while squeezing the other half by the same amount. Finally, we connected the segments in the original order to generate the augmented sample, denoted as $\tilde{x}\left(t\right)$. In the experiment, (w,r)=(1,5) and (w,r)=(3,5) were used with the same probability.
- Random masking: It randomly sets *c*% of the ECG sample to a fixed value. In the experiment, the masking parameter was set to the typical value c=20.

Each augmentation method was pre-trained under the same conditions as MLMCL. Table 5 shows the results of different data augmentation methods on external test sets. In all downstream tasks with a single data augmentation for pre-training, it can be seen that there are different suitable augmentation methods on different test sets. Generally, T-wave masking shows the best single augmentation result on AFDB, while vertical flipping obtains the second-best result. In the task of LTAFDB, vertical flipping showed the best result. As for domain knowledge augmentation, it achieved the best performance on AFDB. Although its performance on LTAFDB did not surpass vertical flipping, it also showed competitive performance. In other words, domain knowledge augmentation improved the quality of representations. This improvement may be due to the fact that T-wave masking and vertical flipping generate more diverse hard samples, which are sufficiently different from the original samples but retain the key diagnostic information. T-wave masking specifically modifies the non-diagnostic region, making the model pay more attention to the features of the P wave and QRS wave in the AF diagnostic region. Vertical flipping modifies the morphology of the P wave and QRS wave but does not affect the rhythm information. Existing studies [42] have shown that positive samples with large morphological differences but the same rhythm can better extract rhythm invariance. In addition, vertical flipping will not cause confusion between the P wave and f wave. These changes force the model to learn more discriminative and robust representations.

#### 4.4.2. Contrastive Loss

The ablation studies on different contrastive losses were evaluated on the AFDB and LTAFDB datasets. We examined the effectiveness of each contrastive loss and gradually combined each contrastive loss pairwise and finally combined all types of contrastive losses. Table 6 shows the ablation experiment results of six combination variants. Here, **T**, **C**, and **L** represent temporal contrastive loss, channel contrastive loss, and label contrastive loss, respectively. Among them, variant **T** only performs temporal contrastive learning on the intermediate hidden representations, thus updating only the convolutional feature extraction network in the encoder. The variant **L** indicates that only label CL is performed, so only labeled data are used and unlabeled data are not used. It should be noted that the equal λ value is used for different contrast losses across variants, whether labeled data or unlabeled data.

All variant experiments used domain knowledge augmentation and maintained the same training conditions as the MLMCL method. For the case of using only a single contrastive loss (**T**, **C**, or **L**), different optimal methods were obtained for different test sets, because each test task has a specific training dataset and data distribution. For the combination of two contrastive losses, it can be seen that on the AFDB database, the combinations of two contrastive losses achieved better Acc and F1 than using a single loss. On the LTAFDB database, only the **C** + **L** combination obtained better performance than using **C** or **L** alone. In addition, it is worth noting that the MLMCL method represented by the combination **T** + **C** + **L** achieved competitive or the best performance on all external test datasets, which means that the proposed MLMCL method is quite robust.

#### 4.4.3. No Projecter

**Figure 7:**
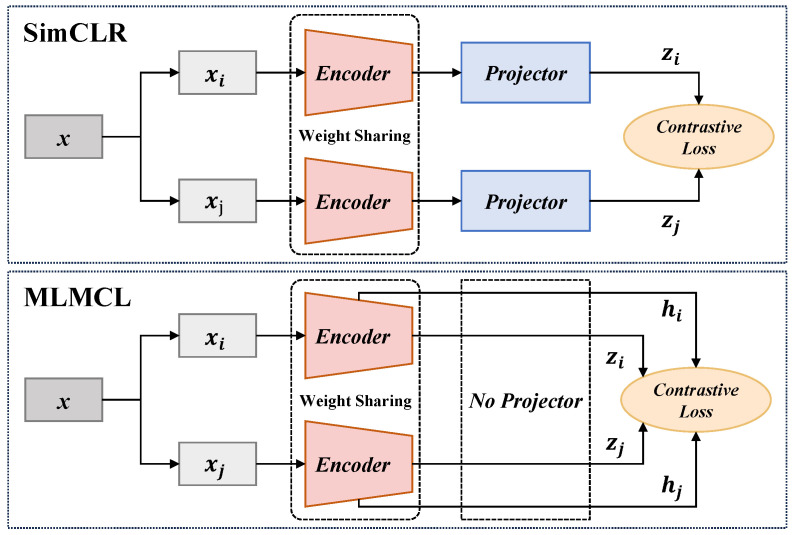
The comparison between MLMCL and SimCLR.

As shown in Figure 7, most CL-based methods employed a projector setup similar to SimCLR to achieve better performance for downstream tasks. They adopted a projector in pre-training to project the encoder representations into the latent space to calculate CL loss and discarded the projector in the downstream tasks. Differently, the proposed MLMCL did not set an additional projector and simultaneously conducts contrastive learning on the encoder representations and the intermediate hidden representations. To explore the necessity of the projector in MLMCL, we conducted the ablation experiment of the projector. The projector was set as a two-layer MLP, and its output size was kept the same as the input size. Table 7 shows the AF detection results of the proposed method with and without a projector. It can be seen that having a projector did not achieve better performance on either the AFDB or LTAFDB database. Considering that the projector may reduce the performance, MLMCL did not use a projector for pre-training, which can also reduce the computational burden.

### 4.5. Comparison with Existing Methods

In addition to the contrastive learning methods [36] for AF detection, there are also many studies on AF detection that adopted other strategies. However, few studies have reported cross-dataset test results, and some of them have used different training and testing datasets from those in this paper, which limits the number of studies available for comparison. Therefore, in order to achieve a good overall comparison with the cross-dataset evaluation results of other studies, we adjusted to use the same labeled training set and testing set for pre-training, fine-tuning, and testing. The comparison results with existing studies are shown in Table 8. Regarding the AF detection task, the method proposed in this paper outperformed most existing methods in different metrics, including Acc and F1. Compared to existing studies that only use labels for supervised learning, MLMCL simultaneously utilizes labels and the ECG inherent consistency to obtain higher-quality representations. In addition, we guided and optimized the pre-training according to the downstream AF detection task. We introduced domain knowledge augmentation for AF and used downstream labels for semi-supervised learning, which reduced the gap between the pre-trained representations and the requirements of downstream tasks, thereby achieving better performance.

## 5. Conclusions

In this work, we proposed a semi-supervised contrastive learning method, MLMCL, for AF detection, aiming to bridge the gap between the limited availability of labeled data in AF detection of single-lead ECG and the requirements for model robustness and generalization. MLMCL utilizes multi-level feature representations of the encoder to perform multiple contrastive learning, fully exploiting the temporal consistency, channel consistency, and label consistency. This method simultaneously uses both labeled and unlabeled data for pre-training to obtain the robust features required for downstream tasks, achieving better performance with limited labels and reducing the dependence on ECG labels. In addition, by utilizing the ECG diagnostic knowledge related to AF, a domain knowledge augmentation method was proposed to generate hard samples that would be sufficiently different from the original samples, enabling a full learning of distinguishable representations. We verified the stability and generalization of the MLMCL method on multiple different external data sets in a challenging cross-dataset testing environment. The abundant baseline settings and extensive ablation studies demonstrate that the proposed MLMCL effectively and robustly outperforms other existing AF detection methods. Our future work aims to test our method on more data sets to more fully examine its generalization ability. This method can be easily extended to the scenario of AF detection in multi-lead ECG signals and has reference significance for the development of other arrhythmia contrastive learning methods.

## Figures and Tables

**Table 1:** Details of the datasets used in this study.

Dataset	Freq	NR	NS	Lead	Record Length	Rhythms	TD	AFD	NEB
AFDB	250 Hz	25	25	ECG1, ECG2	10 h	4	234.28 h	93.40 h (39.87%)	/
CPSC2021	200 Hz	1436	105	I, II	0~6.8 h	3	480.19 h	164.44 h (34.24%)	93,545 (4.4%)
LTAFDB	128 Hz	84	84	ECG1, ECG2	6~26 h	9	1960.60 h	1030.89 h (52.58%)	285,100 (3.2%)
MITDB	360 Hz	48	47	II, V1, V2, V4, V5	0.5 h	15	24.07 h	2.21 h (9.18%)	34,442 (31.5%)

Freq: Sampling frequency, NR: Total number of records, NS: Number of subjects in the recording, TD: Total duration, AFD: AF duration, NEB: Number of ectopic beats.

**Table 2:** Data description after segmentation.

Dataset	Segment Length	Overlap	Non-AF Segments	AF Segments
AFDB	30 s	15 s	31,547	21,383
CPSC2021	30 s	0 s	68,904	37,132
LTAFDB	30 s	0 s	100,163	115,952
MITDB	30 s	15 s	4579	452

**Table 3:** Linear probing results of different methods on the external ECG datasets.

	AFDB				LTAFDB			
Methods	Acc (%)	Pre (%)	Sen (%)	F1 (%)	Acc (%)	Pre (%)	Sen (%)	F1 (%)
FS	50.64	47.63	47.75	47.56	63.07	63.99	63.71	62.99
FS + DA	47.39	44.03	44.34	44.06	59.15	61.15	60.37	58.53
T-S	67.12	69.10	61.20	59.84	62.71	69.14	64.59	61.00
SimCLR	80.63	81.77	81.31	80.61	81.70	83.58	78.72	79.81
MLMCL	98.18	98.33	97.90	98.10	96.61	96.56	96.64	96.60

**Table 4:** Full fine-tuning results of different methods on the external ECG datasets.

	AFDB				LTAFDB			
Methods	Acc (%)	Pre (%)	Sen (%)	F1 (%)	Acc (%)	Pre (%)	Sen (%)	F1 (%)
FS	89.98	90.85	88.47	89.32	95.88	95.82	95.92	95.86
FS + DA	95.03	95.70	94.11	94.76	95.82	95.75	95.90	95.81
T-S	93.65	94.65	92.40	93.25	96.24	96.18	96.28	96.22
SimCLR	96.55	96.41	96.43	96.42	95.68	95.61	95.78	95.67
MLMCL	99.14	99.19	99.03	99.10	97.34	97.34	97.31	97.33

**Table 5:** Ablation study of data augmentation.

	AFDB				LTAFDB			
Data Augmentation	Acc (%)	Pre (%)	Sen (%)	F1 (%)	Acc (%)	Pre (%)	Sen (%)	F1 (%)
Gaussian Noise	96.56	95.90	95.57	95.73	96.89	96.85	96.91	96.88
Channel Scaling	96.04	95.56	94.60	95.08	97.40	97.38	97.39	97.38
Baseline Wander	97.55	97.72	96.19	96.95	96.32	96.26	96.36	96.31
Horizontal flipping	97.73	97.84	96.52	97.17	96.23	96.16	96.30	96.21
Time Warping	97.88	98.17	96.56	97.35	97.05	97.02	97.04	97.03
Random Masking	97.79	97.65	97.76	97.71	96.70	96.64	96.74	96.68
Vertical Flipping	98.82	98.54	98.54	98.54	**97.44**	**97.43**	**97.43**	**97.43**
T-wave Masking	99.10	99.14	98.99	99.10	96.85	96.80	96.88	96.84
MLMCL	**99.14**	**99.19**	**99.03**	**99.10**	97.34	97.34	97.31	97.33

**Table 6:** Ablation study of multiple contrastive loss.

	AFDB				LTAFDB			
Methods	Acc (%)	Pre (%)	Sen (%)	F1 (%)	Acc (%)	Pre (%)	Sen (%)	F1 (%)
**T**	97.90	98.02	97.63	97.81	96.44	96.40	96.46	96.43
**C**	98.35	98.56	98.04	98.28	96.21	96.14	96.30	96.20
**L**	96.81	96.60	96.78	96.70	96.65	96.61	96.56	96.87
**T + C**	98.47	98.66	98.18	98.41	95.79	95.71	95.90	95.77
**T + L**	98.23	98.31	98.01	98.15	95.96	95.90	95.99	95.94
**C + L**	**99.18**	**99.23**	**99.07**	**99.15**	97.00	96.97	97.02	96.99
MLMCL	99.14	99.19	99.03	99.10	**97.34**	**97.34**	**97.31**	**97.33**

**Table 7:** Ablation study of the proposed method with/without a projector.

Test Set	Methods	Acc (%)	Pre (%)	Sen (%)	F1 (%)
AFDB	With a projector	99.04	99.09	98.92	99.00
	Without a projector	99.14	99.19	99.03	99.10
LTAFDB	With a projector	96.69	96.63	96.72	96.67
	Without a projector	97.34	97.34	97.31	97.33

**Table 8:** Comparison of the classification performance between the proposed method and previous works.

Study	Year	Training Set	Test Set	Acc (%)	Pre (%)	Sen (%)	F1 (%)
Andersen et al. [54]	2019	AFDB	MITDB	87.40	45.45	98.96	/
Shi et al. [55]	2020	AFDB	MITDB	87.4	**81.11**	**97.46**	/
Seo et al. [56]	2021	AFDB	MITDB	86.68	/	/	/
Liu et al. [57]	2022	AFDB	MITDB	92.23	53.92	95.17	68.84
MLMCL	2024	AFDB	MITDB	**94.31**	80.62	96.87	**86.37**
Wen et al. [58]	2022	CPSC2021	LTAFDB	/	94.60	89.50	91.98
Yun et al. [59]	2024	CPSC2021	LTAFDB	/	96.45	94.84	95.64
MLMCL	2024	CPSC2021	LTAFDB	**97.34**	**97.34**	**97.31**	**97.33**

## Data Availability

The CPSC2021, AFDB, LTAFDB, and MITDB datasets are publicly available at https://physionet.org/content/cpsc2021/1.0.0/, https://physionet.org/content/afdb/1.0.0/, https://physionet.org/content/ltafdb/1.0.0/ and https://physionet.org/content/mitdb/1.0.0/, respectively.
